# HiCPotts: an R/bioconductor package to identify significant interactions in chromosome conformation capture data and model sources of bias

**DOI:** 10.1093/bioinformatics/btag673

**Published:** 2026-09-10

**Authors:** Itunu Godwin Osuntoki, Andrew Harrison, Hongsheng Dai, Yanchun Bao, Nicolae Radu Zabet

**Affiliations:** Statistics, Modelling and Economics Department, UK Health Security Agency, London, NW9 5EQ, United Kingdom; School of Mathematics, Statistics and Actuarial Science, University of Essex, Colchester, CO4 3SQ, United Kingdom; School of Mathematics, Statistics and Actuarial Science, University of Essex, Colchester, CO4 3SQ, United Kingdom; School of Mathematics, Statistics and Actuarial Science, University of Essex, Colchester, CO4 3SQ, United Kingdom; School of Mathematics, Statistics and Physics, Newcastle University, Newcastle upon Tyne, NE1 7RU, United Kingdom; School of Mathematics, Statistics and Actuarial Science, University of Essex, Colchester, CO4 3SQ, United Kingdom; Blizard Institute, Barts and The London School of Medicine and Dentistry, Queen Mary University of London, London, E1 2AT, United Kingdom; Centre for Epigenetics, Queen Mary University of London, London, E1 2AT, United Kingdom

## Abstract

**Motivation:**

Chromosome Conformation Capture methods, including Hi-C, micro-C or Capture-C, are used to map chromatin interactions genome-wide. Most of the existing computational methods do not account for sources of bias (such as DNA accessibility, GC content or TE content) in the data.

**Results:**

We previously developed ZipHiC, a Bayesian method based on the hidden Markov random field (HMRF) model and the Approximate Bayesian Computation (ABC), that uses zero-inflated Poisson distribution to model the noise, signal and false signal of the data and showed that this approach was able to detect bias from DNA accessibility, GC content and TE content in both Hi-C and micro-C data. Here, we present HiCPotts, another Bayesian method based on the HMRF model and the ABC that uses a zero-inflated Negative Binomial distribution instead to model the noise and signal of the data. We systematically show that HiCPotts reduces false positives and increases recovery of true interactions compared to ZipHiC, but also compared to other methods such as FastHiC, Juicer and HiCExplorer. Most importantly, we provide an R/Bioconductor package that allows modelling the noise, signal and false signal using various distributions such as the zero-inflated Negative Binomial (ZINB) and the zero-inflated Poisson distribution (ZIP).

**Availability and implementation:**

https://bioconductor.org/packages/HiCPotts/

## 1 Introduction

The genome folds in the nucleus ensuring that distal regulatory elements contact the correct promoters across kilobases to megabase distances Bonev and Cavalli (2016). Association between distant regions of the genome has been detected by several chromatin capture methods techniques (including Hi-C or micro-C), which allowed building 3D contact maps of the entire genome in individual cells, tissues or developmental stages Lieberman-Aiden *et al.* (2009); Hsieh *et al.* (2015).

Advances in computational methods have progressively improved the spatial resolution of Hi-C and micro-C data Goel *et al.* (2023). Hi-C and micro-C produce large, sparse contact-count matrices and the data can be influenced by technical and biological factors. Fragment length, GC content, and mappability are major Hi-C bias Yaffe and Tanay (2011). Iterative Correction and Eigenvector decomposition (ICE) balances rows and columns to equalise visibility and remove such bias Imakaev *et al.* (2012), but assumes all loci are equally observable and becomes computationally expensive at high resolution Imakaev *et al.* (2012). Knight-Ruiz matrix balancing provides a faster alternative by scaling rows and columns so their sums equal one, yielding a doubly stochastic matrix Knight and Ruiz (2013).

More recent methods model bias within explicit statistical frameworks. Hi-C-DC uses distance-stratified negative binomial regression, incorporating GC content, mappability, and fragment length to assess interaction significance Carty *et al.* (2017). Hi-C-DC+, an extension of Hi-C-DC, improves preprocessing, distance-stratified background modelling, and differential analysis, increasing sensitivity and interpretability Sahin *et al.* (2021). However, both Hi-C-DC and Hi-C-DC+ treat each interaction independently and ignore spatial dependencies and neighbourhood structure Sahin *et al.* (2021).

Other recent approaches include AutoHiC, which uses a deep neural network to jointly learn normalisation weights and correct assembly errors, accounting for bias but still treating contacts independently and omitting spatial correlations Jiang *et al.* (2024). HiC2Self, a self-supervised denoising method, improves Hi-C data quality by learning latent structure without labels, but likewise lacks explicit modelling of genomic covariates or neighbourhood dependencies Yang *et al.* (2026). Despite all these advances, most methods still classify contact counts into “signal” versus “background” and treat each contact as independent, neglecting the strong two-dimensional neighbourhood dependencies that characterize contact maps.

One of the ways to address spatial dependence is by using the Bayesian hidden-Markov random-field (HMRF) model. The HMRFBayesHiC method captures spatial dependencies via a hidden Markov random field, offering a principled Bayesian framework that accounts for genomic bias and neighbourhood correlations, therefore making it an early precursor to spatial models like ZipHiC Osuntoki *et al.* (2022); Xu *et al.* (2016a). ZipHiC extended on the HMRFBayesHiC by employing the Potts model in modelling spatial dependency directly on the contact matrix Osuntoki *et al.* (2022). It treats each pixel in the Hi-C matrix as part of a 2D lattice and encourages adjacent loci to share similar latent states, enabling borrowing of information across rows and columns. ZipHiC calculates the Potts normalising constant with an Approximate Bayesian Computation (ABC) sampler because otherwise it is computationally intractable. ZipHiC further classified contact counts into multiple components (“noise,” “signal” and “false signal”) and reduced false positives relative to other methods. However, it models the noise using a zero-inflated Poisson distribution, which might not accurately represent the noise in genomics data.

Here, we present a similar statistical approach to detect biologically significant chromatin interactions, using the zero-inflated Negative Binomial instead of zero-inflated Poisson distribution. The assumption of zero-inflated Negative Binomial distribution allows for improvement in the identification of each component by taking into consideration overdispersion that might occur within the components. We performed systematic analysis on real data, which shows improved performance over existing methods. Finally, we make this method available as an R/Bioconductor package called HiCPotts https://bioconductor.org/packages/HiCPotts/ that allows modelling of the noise using either the zero-inflated Negative Binomial or the zero-inflated Poisson distribution.

## 2 Materials and methods

### 2.1 HiCPotts model

We previously described a similar model using the zero-inflated Poisson distribution to model the noise and signal in Osuntoki *et al.* (2022). Here, we improved the model and assumed the zero-inflated Negative Binomial distribution. Beyond the methodological extension introduced here, HiCPotts retains the Potts–HMRF spatial prior, ABC sampling framework, and three-component structure used in ZipHiC. An additional practical contribution is the implementation of HiCPotts as a fully documented R/Bioconductor package, whereas ZipHiC was distributed as a collection of scripts.

#### 2.1.1 Notations

Using the contact matrix between pairs of bins generated from Hi-C experiments, let $y_{ij}$ denote the observed contact frequency between bin *i* and bin *j* in *N* total bins, and $D_{ij}$ denote the genomic distance between bin *i* and bin *j*. Let $GC_{ij}$ denote the average percentage of Guanine and Cytosine, $TE_{ij}$ denote the average number of transposable elements (TEs), and $ACC_{ij}$ denote the average DNA accessibility score in bins *i* and *j*. For convenience and readability, we denote the interaction pair of bins *i* and *j* as s={i,j} and also $D_{s}$, $GC_{s}$, $ACC_{s}$ and $TE_{s}$ to denote the observation value for interaction *s*.

#### 2.1.2 Mixture model for data

We assume that the contact frequency density of the $y_{ij}$ comes from the following mixture model of *K* components, and the first component is a zero-inflated Negative Binomial distribution (*ZINB*) which represents the noise (see below), and the other components are modelled using the Negative Binomial (*NB*) distribution:


(1) $$\begin{array}{cc} f(y_{ij})=\alpha_{1}\text{ZINB}(y_{ij}|\lambda_{ij}^{\left(1\right)},\omega^{\left(1\right)},\tau^{\left(1\right)}) & \\ \;+\sum_{k=2}^{K}\alpha_{k}\text{NB}(y_{ij}|\lambda_{ij}^{\left(k\right)},\omega^{\left(k\right)}), \end{array}$$

where


(2) $$\text{ZINB}(y_{ij}|\lambda_{ij},\omega ,\tau )=\{\begin{array}{cc} \tau +(1-\tau )\text{NB}(y_{ij}|\lambda_{ij},\omega ), & \text{if}\;y_{ij}=0,\\ (1-\tau )\text{NB}(y_{ij}|\lambda_{ij},\omega ), & \text{if}\;y_{ij}>0, \end{array}$$

τ is the probability of extra zeros, $\lambda_{ij}$ is the mean of the *k*th component, ω is the overdispersion parameter of the *k*th component. $\alpha_{k}$ is the unknown percentage of *k*th component subject to the constraint $\sum_{k=1}^{K}\alpha_{k}=1$. Since ω controls the degree of overdispersion, as ω→∞ the equation approaches a Poisson (λ) model. A smaller value of ω<1 implies that we have a case of overdispersion Dai *et al.* (2013).

The above mixture model can be interpreted within a latent variable framework. We introduce the latent variable $z_{ij}\in \{1,2,\ldots ,K\}$, where $z_{ij}=k$ indicates that $y_{ij}$ follows the distribution of component *k*. Furthermore, $\lambda_{ij}^{\left(k\right)}$ denotes the mean interaction between fragments *i* and *j* when it is from the *k*th component. Increasing the number of components *K* enhances the flexibility of the framework, allowing it to model diverse scenarios of interaction patterns. However, *K* is fixed at three components in the current implementation, because of biological interpretation and also chosen to mirror the noise/signal/false-signal decomposition of ZipHiC.

Because the Hi-C (or micro-C) contact maps exhibit an excess of zero-counts and unequal mean-variance relationships, we assume that the noise follows a ZINB distribution rather than a ZIP distribution. Specifically, the ZINB distribution has mean (1−τ)λ and variance $(1-\tau )\left(\lambda +\frac{\lambda^{2}}{\omega }\right)+\tau (1-\tau )\lambda^{2}$. In addition, we corrected the sources of bias by modelling $\lambda_{s}^{\left(k\right)}$ with s={i,j},k=1,2,…,K as


(3) $$\begin{array}{c} \;\text{log}(\lambda_{s}^{\left(k\right)})=\beta_{0}^{\left(k\right)}+\beta_{1}^{\left(k\right)}\;\text{log}(D_{s})+\beta_{2}^{\left(k\right)}\;\text{log}(GC_{s})\\ +\beta_{3}^{\left(k\right)}\;\text{log}(TE_{s})+\beta_{4}^{\left(k\right)}\;\text{log}(Acc_{s}) \end{array}$$

The unknown model parameters are denoted as $\Theta =(\tau ,\omega ,\beta^{\left(k\right)},k=1,\ldots ,K)$ and the probability mass function for the observed data y, given the latent variables z, is denoted as f(y|Θ,z).

To prevent label switching and preserve a consistent interpretation of mixture components across MCMC chains, we resolve component identifiability without imposing a hard ordering constraint during sampling. We define $\mu^{(k)*}=\text{exp}(\beta_{0}^{\left(k\right)})$ as the baseline mean contact frequency of the *k*-th component. Component 1 (noise) is structurally distinct; it alone carries the zero-inflation term and is never reordered. Components 2 and 3 are exchangeable during sampling; rather than enforcing a fixed order $\mu^{(2)*}<\mu^{(3)*}$ in the prior, we add a soft, symmetric penalty between them that discourages the chain from lingering near a label tie (the mechanism behind label-switching) without forbidding it from crossing, so the data can still determine which ordering is preferred. After sampling, we assign the labels “noise,” “signal” and “false signal” post hoc for the whole chain, ordering components 2 and 3 by their post-burn-in mean baseline intensity $\mu^{(2)*}<\mu^{(3)*}$ whenever their intercepts’ 95% credible intervals are disjoint. When those intervals overlap, intensity alone cannot distinguish the two components in that fit, and we instead break the tie by relative population size, recording which criterion was used. This procedure yields consistent component labels across chains and improves interpretability, but because the “signal” and “false signal” identities are assigned by this convention rather than fixed in the model, users should still verify, particularly under a population tie-break or when the two components’ intensities are close; which component was labelled signal in their own fit rather than assuming it by index.

#### 2.1.3 Potts model

To capture the spatial dependence, our method employs a Hidden Markov Random Field (HMRF), which extends the widely known standard Hidden Markov Model (HMM). The HMRFs are widely applied across disciplines such as image analysis (Zhang *et al.* 2001), gene expression data (Wei *et al.* 2008), and a population genetics study (François *et al.* 2006). Adapting the Potts model (Wu, 1982), a Markov random field-based method that provides a flexible way to model spatially dependent data, the prior distribution for the latent variable $z_{s}$ is expressed as a function of its neighbours in a 2D grid. The latent variable z is written as


(4) $$p(z|\gamma )=\frac{1}{C(\gamma )}\text{exp}(\gamma \sum_{\left(s∼t\right)}\delta_{z_{s}z_{t}})$$

where $\delta_{z_{s}z_{t}}$ is the Kronecker symbol which takes the value 1 when $z_{s}=z_{t}$ and 0 otherwise. *t* denotes the neighbouring fragment pairs of *s*, i.e. s∼t means *s* and *t* are neighbours in the Hi-C matrix. Our set of latent variables $z_{ij}=z_{s}$ depends on the status of the neighbors of *s*, $N_{s}=\{(i+1,j),(i-1,j),(i,j+1),(i,j-1)\}$. The neighbouring $\sum_{\left(s,t\right)}\delta_{z_{s}z_{t}}$ can be defined as the sum of the influence of neighbours of *s* controlled by a non-negative interaction parameter γ. When γ is 0, the latent variables behave independently. Higher values of γ, such as γ=1, encourage spatial consistency, such that neighbouring bins are more likely to be placed in the same component. The normalizing constant term C(γ), also known as the partition function, is written as


(5) $$C(\gamma )=\sum_{z}\;\text{exp}\;(\gamma \sum_{\left(s∼t\right)}\delta_{z_{s}z_{t}})$$

where $\sum {\;}_{z}$ denotes the summation over $z_{s}$ at all interactions *s* and it depends on the interaction parameter γ. The normalising constant term is computationally intractable in higher orders; therefore, methods such as the Approximate Bayesian Model (ABC) (Beaumont *et al.* 2002), a likelihood-free approach, can be used.

#### 2.1.4 Approximate Bayesian Computation (ABC)

Given an observed dataset $Y=(y_{1},y_{2},\ldots ,y_{n})$ that is associated with the models in Equations (1) to (4), the ABC algorithm (Beaumont *et al.* 2002) used here is written as follows:

- Initialise parameters Θ and latent variables z and then repeat the following;
  1. Simulate an new value $\gamma^{*}$ from the prior distribution $\pi_{0}(\gamma )$;
  2. Generate all latent variables $z_{ij}$ from (4);
  3. Simulate a pseudo data set $y^{*}$ from f(y|Θ,z);
  4. Compute the distance (absolute or euclidean) *d* between the simulated data and the observed data;
  5. fix a tolerance ϵ or use an empirical quantile of $d(y^{*},y)$ which often corresponds to 1% quantile (Beaumont *et al*. 2002)
  6. Accept $\gamma^{*}$ if the distance is less than ϵ, otherwise reject and start from step 1 again.
  7. Generate a parameter value Θ from the posterior distribution $\pi_{0}(\Theta )f(y|\Theta ,z)$;

#### 2.1.5 Bayesian inference

A Bayesian framework is adopted, where the posterior distribution is proportional to the product of the prior and likelihood. Also, an Empirical Bayes approach is used to define a hierarchical prior structure, with hyper-parameters estimated from the data itself, thereby reducing sensitivity to prior mis-specification.

Each of the model parameters Θ and the interaction parameter γ is assigned its own prior $\pi_{0}(\cdot )$, whose conditional posterior form is derived in the *Supplementary Material* (equations S3–S11, available as supplementary data at *Bioinformatics* online) and used within the Metropolis-within-Gibbs and ABC samplers (Supplementary Material, available as supplementary data at *Bioinformatics* online, Algorithms 1–2). Specifically: the prior for $\beta^{\left(k\right)}$ in Equation (3) is Normal, with hyperparameters either fixed by the user, or, under the empirical (data-driven) setting, re-estimated at every MCMC iteration from the subset of bin-pairs currently assigned to component *k* under the latent state z (Supplementary Material, equations S5–S6, available as supplementary data at *Bioinformatics* online); because this re-estimation uses the same data as the likelihood, it is an iterated, hierarchical form of empirical Bayes; the zero-inflation parameter τ is assigned a uniform Beta(1,1) prior, updated at each iteration via a conjugate Beta-Bernoulli Gibbs step (Supplementary Material, equation S3, available as supplementary data at *Bioinformatics* online) using the current counts of bin-pairs assigned or not assigned to the noise component; the overdispersion parameters $\omega^{\left(k\right)}$ are each assume to follow the Gamma prior, independent of whether the empirical or fixed prior is used for β (Supplementary Material, equations S7–S8, available as supplementary data at *Bioinformatics* online); the Potts interaction parameter γ is assigned a Beta(2,2) prior, used within the ABC step (Supplementary Material, equation S11, available as supplementary data at *Bioinformatics* online and Algorithm 1); and the mixture weights $\alpha_{k}$ in Equation (1) are not estimated as free parameters. A constant prior contribution $\pi_{0}(\alpha )\propto 1$ is assumed, so no explicit $\pi_{0}(\alpha )$ term appears in the model. Instead, the realised proportion of bin-pairs assigned to each component is induced by the interaction between the Potts spatial prior in Equation (4) and the component-specific likelihood acting on the latent allocation field z. This proportion can be recovered post hoc as the empirical fraction of sites for which $z_{s}=k$.

Further to the use of the Empirical Bayes approach, we also adopt the conventional Bayesian approach. For the conventional Bayesian approach, the prior for the parameter β follows the normal distribution. For example, $\beta_{0}^{\left(1\right)}∼N$($\beta_{0}^{\left(1\right)};5,1$), γ∼β(γ;5,5) and $\pi_{0}$ set to 0.5. See *Results* and *Supplementary Material*, available as supplementary data at *Bioinformatics* online.

In this paper, we convert posterior means into component assignments using a fixed posterior-probability threshold: an interaction *s* is declared significant if $P(z_{s}=2(\mathrm{signal})|y)\geq 0.90$.

#### 2.1.6 Running time

We benchmarked ZIP and ZINB in HiCPotts on simulated data of increasing size (single CPU core, R 4.5.0): ZINB was 18%–58% slower per iteration, narrowing with region size, and a 1000-bin (10 Mb, 10 Kb) chain at 20 000 iterations took ∼1.7–2.0 days. FastHiC, also HMRF-based, shows the same quadratic runtime scaling and averages ∼7 minutes per topological domain (Xu *et al.* 2016b); Juicer and HiCExplorer instead use deterministic matrix-balancing and observed/expected tests rather than iterative sampling, completing in minutes, but none of the three jointly models spatial dependency, overdispersion and covariate bias as HiCPotts does.

Given the computational cost of the MCMC and ABC sampling method, we focused our analyses on a subregion of the human genome, up to 10 Mb for the human chromosome 8 analyses, and a subregion of the Drosophila genome, on chromosome 2 L, rather than whole-chromosome or genome-wide maps.

### 2.2 Datasets and preprocessing

#### 2.2.1 Drosophila dataset

Model performance was evaluated using the Hi-C map of Kc167 cell lines in *Drosophila* from (Eagen *et al.* 2017). Raw data were downloaded and preprocessed with HiCExplorer following the set of parameters from (Chathoth *et al.* 2022, Chathoth and Zabet 2019), with each pair of PE reads aligned separately to the *Drosophila melanogaster* (dm6) genome (dos Santos *et al.* 2015) using BWA-mem (Li and Durbin 2010) (with options -t 20 -A1 -B4 -E50 -L0). HiCExplorer was then used to build and correct the contact matrices and detect enriched contacts (Ramirez *et al.* 2018) at 2 Kb bins resolution. The matrices were then exported in text format to be loaded in R.

We used DNaseI-seq data from (Kharchenko *et al.* 2011) for DNA accessibility in *Drosophila* Kc167 cells data, and FlyBase (dos Santos *et al.* 2015) for TE annotation in *Drosophila*.

#### 2.2.2 Human datasets

Hi-C and micro-C datasets from H1-hES cells from (Krietenstein *et al.* 2020) were also analysed, following the same preprocessing pipeline used for the *Drosophila* dataset. We also aligned each pair to the human genome hg38 (Schneider *et al.* 2017) using BWA-mem (Li and Durbin, 2010). Contact matrices were constructed using HiCExplorer at 10 Kb resolution. HiCExplorer was also used to detect the enriched contacts (Ramirez *et al.* 2018).

In addition, DNaseI-seq were used to access DNA accessibility from the ENCODE consortium (Thurman *et al.* 2012), with TE annotation sourced from RepeatMasker (http://www.repeatmasker.org). For gene and TE annotations, we used UCSC genome browser annotations Casper *et al.* (2026), while for enhancer annotations, we used Enhancer 2.0. Gao and Qian (2020).

### 2.3 Comparison to other tools

In this paper, we compare our new method, HiCPotts using the Zero-Inflated Negative-Binomial to *(i)* ZipHiC Osuntoki *et al.* (2022), *(ii)* FastHiC (Xu *et al.* 2016b), *(iii)* HiCExplorer (Ramirez *et al.* 2018) and *(iv)* Juicer (Durand *et al.* 2016). For enriched interactions detection, a JAVA implementation of FastHiC was applied using expected counts with values estimated by the HiCExplorer (Ramirez *et al.* 2018).

Then, HiCExplorer was run to generate the matrices, which are then corrected using the following values: (i)  [−1.8,5.0] for Hi-C in Kc167 cells, (ii)  [−2.4,5.0] for Hi-C in H1-hES cells and (iii)  [−2.0,5.0] for micro-C in H1-hES cells; see Supplementary (Ramirez *et al.* 2018). We generated the enriched contacts from the corrected matrix using hicFindEnrichedContacts tool with the observed over expected method (—method obs/exp) (Ramirez *et al.* 2018).

Furthermore, Juicer was used to generate enriched contacts by calling dump via its built-in tools. Specifically, we adopted the observed over expected method (oe) and Knight-Ruiz normalisation (KR) at 2 Kb resolution for the Hi-C data in Kc167 cells and at 10 Kb resolution for the Hi-C and micro-C data in H1-hES cells (Durand *et al.* 2016).

For the rest of the paper, we use HiCPotts or HiCPotts-ZINB to denote the use of the zero-inflated negative Binomial Distribution in the HiCPotts.

The R package used to perform the analysis can be downloaded from Bioconductor HiCPotts.

## 3 Results

**Figure 1:**
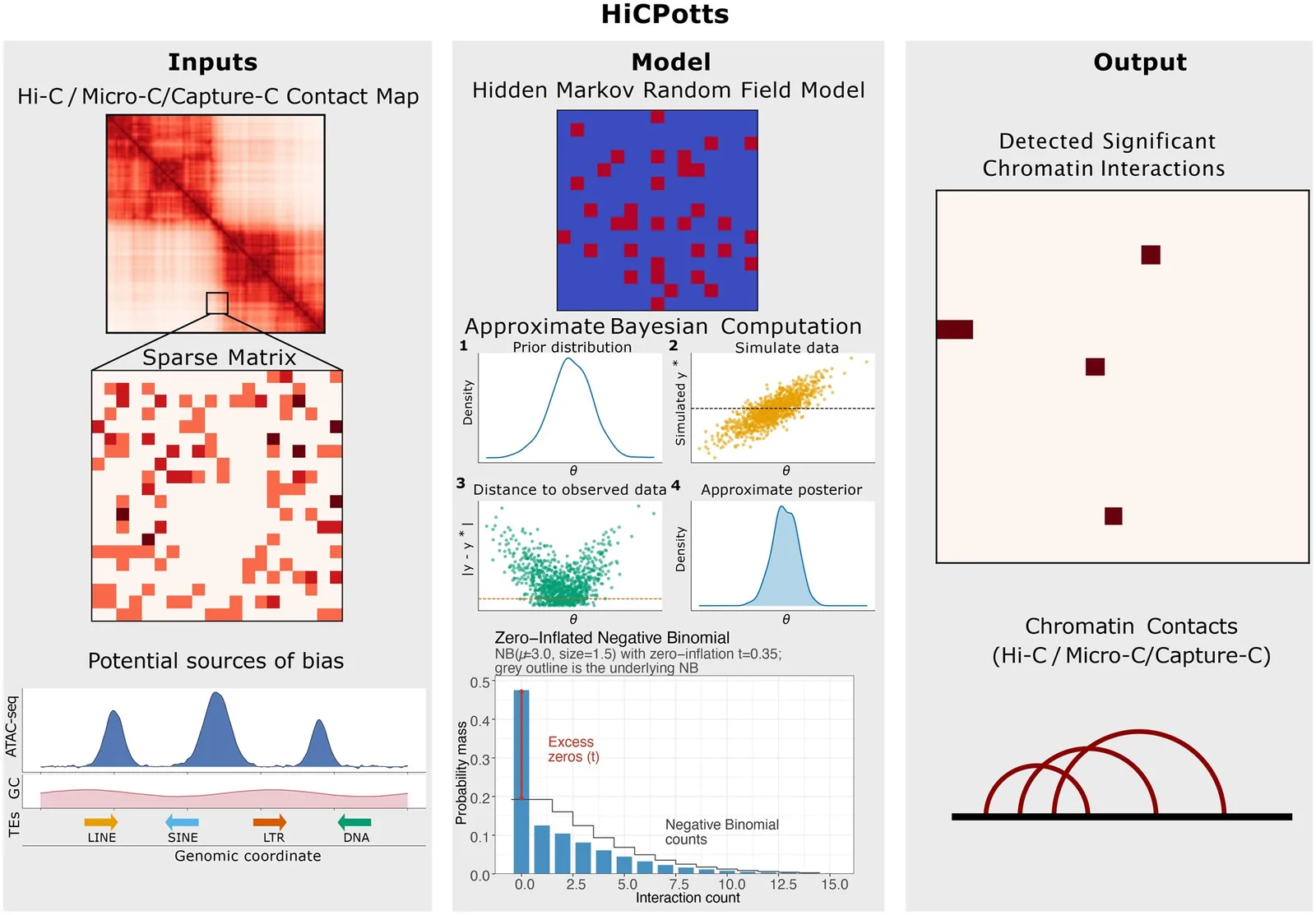
Workflow of HiCPotts.

We developed HiCPotts as an R/Bioconductor package to detect significant interactions from Hi-C, micro-C or Capture-C 3D chromatin data (see Fig. 1). HiCPotts implements a Bayesian method based on the Hidden Markov Random Field HMRF and the Approximate Bayesian Computation ABC and uses a zero-inflated Negative Binomial distribution to model the noise, false signal and signal of the data (see *Materials and Methods*).

### 3.1 Simulated dataset

**Figure 2:**
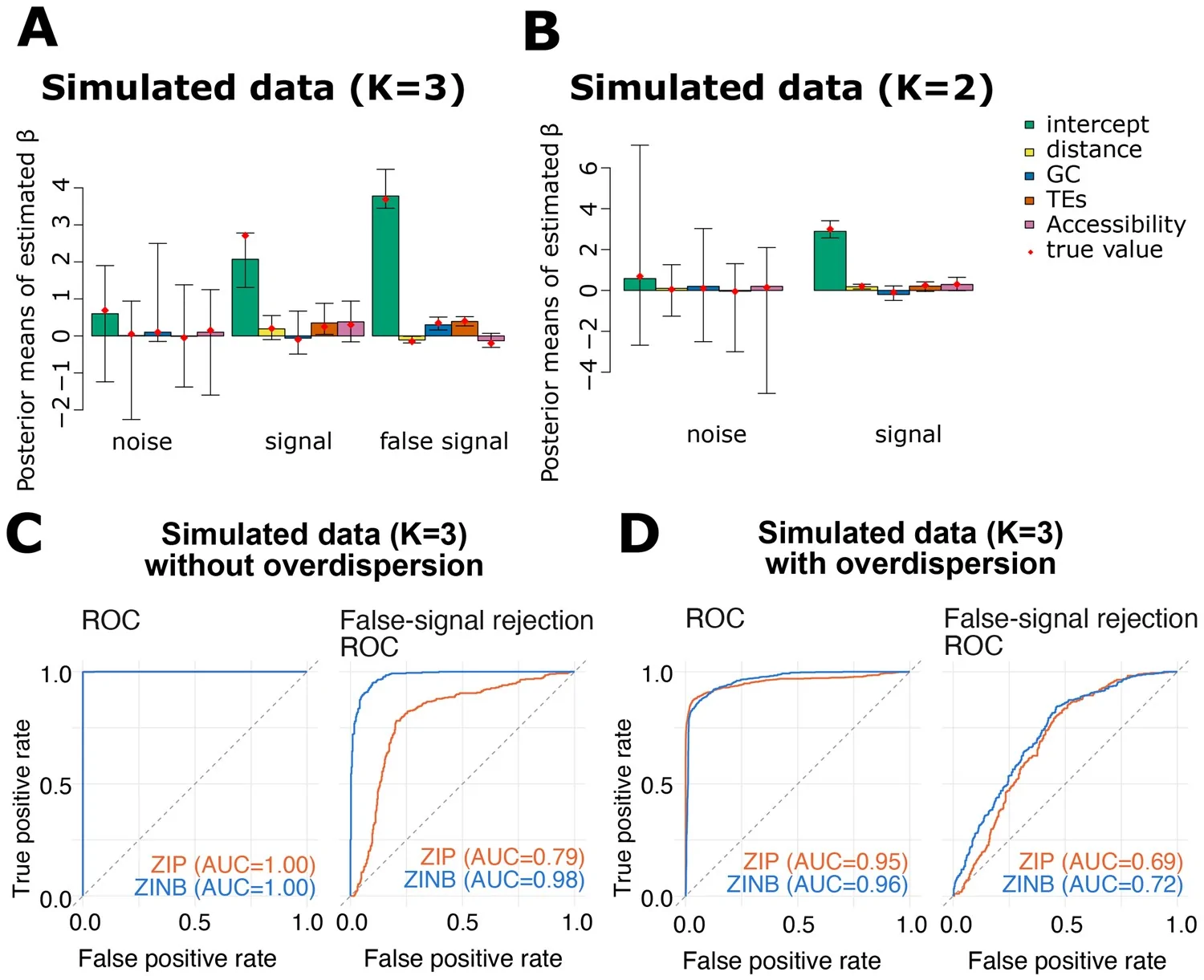
Evaluation of the model with simulated datasets. (A,B) Posterior means of our estimated βs based on the simulated datasets. We plot the estimated values of βs on simulated data with (A) *K* = 3 and (B) *K* = 2. The 95% credible intervals are all shown. (C,D) Evaluation of the predictions based on simulated dataset. We plot Receiver Operator Curves for the false-signal rejection curves for the case of: (C) no overdispersion introduced in the simulated and (D) overdispersion (ω=0.5 for all components) introduced in the simulated data.

Before applying HiCPotts to real data, we first verified that it can estimate the sources of bias from simulated data. We drew the latent state field z from the Potts prior (Equation 4), then generated the component means from the log-linear predictor (Equation 3), and drew counts from the zero-inflated Negative Binomial mixture (Equations 1 and 2). Unless stated otherwise, each scenario used n=400 observations from the mixture model (Equation 1), 15 independently simulated replicates, and 5000 MCMC iterations. We reported the posterior mean estimate and its 95% credible interval, averaged across the 15 replicates; aggregated over all parameters, the 95% interval contained the true value in more than 95% of the replicate-parameter combinations in every scenario reported here. HiCPotts accurately estimates the spatial parameter γ, the zero-inflation parameter τ, and the sources of bias estimates (Fig. 2A).

To evaluate the performance of the method on simulated data, we also included the ROC curves to provide a threshold-independent comparison of the two approaches (Fig. 2C and D). These show that both methods maintain strong discrimination of true interactions, while the relative advantage of ZINB can be seen. Under the no-overdispersion scenario (Fig. 2C), where the data were generated from a Poisson distribution, ZIP performed as well as ZINB. This is expected because ZIP is closely aligned with the underlying Poisson-generating distribution, whereas the additional dispersion flexibility of ZINB is unnecessary in this scenario. When overdispersion was introduced (Fig. 2D), the classification performance of ZIP weakened only slightly ($\mathrm{AURO}C_{\mathrm{ZIP}}=0.95$ compared to $\mathrm{AURO}C_{\mathrm{ZINB}}=0.96$). In addition, we evaluated false-signal rejection since this directly assesses the ability of the models to distinguish the false-signal component from the true-signal component Fawcett (2006). ZINB consistently showed higher false-signal rejection, indicating that it more reliably prevented observations from the false-signal component from being misclassified as true signals (Fig. 2C and D). This further shows the importance of the ZINB distribution in separating true signals from false signals.

Next, we studied sensitivity to the zero-inflation parameter and to the spatial parameter. Across low, moderate and high zero-inflation scenarios, the posterior mean of τ estimated the true value: 0.11 for τ=0.1, 0.36 for τ=0.4 and 0.68 for τ=0.7 (Table 1). We also reported the 95% credible interval of the different τ estimates. The posterior mean of γ also shows the model’s estimate of the true values of the spatial parameter, 0.48 when the true value was 0.2, to 0.57 for 0.6 and 0.79 for 0.9 (Table 2); the upward bias in (γ) under weak spatial specification is considered to be due to weak simulated dataset identification, and approximate inference for the Potts normalising constant and not model misspecification.

**Table 1:** Zero-inflation parameter τ across three true levels (ZINB, 15 replicates each, other true values as in Fig. 2A).

**True** τ	Mean estimate	95% CI
0.1 (low)	0.11	[0.01, 0.21]
0.4 (moderate)	0.36	[0.12, 0.54]
0.7 (high)	0.68	[0.48, 0.85]

**Table 2:** Spatial (γ) estimates across three true levels (ZINB, 15 replicates each, other true values as in Fig. 2A).

**True** γ	Mean estimate	95% CI
0.2 (weak)	0.48	[0.06, 0.89]
0.6 (moderate)	0.57	[0.06, 0.98]
0.9 (strong)	0.79	[0.07, 0.96]

The Finally, we assessed the fitted three-component model when the data-generating process contained only two components, noise and signal. Figure 2B shows that even though our method uses *K* = 3, the estimated parameters closely match the true values, which shows that none of the observations is classified into the false signal component. The γ and τ, together with the dispersion and covariate effects, were recovered accurately. Additional simulation results on overdispersion, noise/signal composition and the Negative Binomial distribution model are provided; see *Supplementary Material*.

### 3.2 Identification of significant interactions in high resolution Hi-C data in *Drosophila melanogaster*

**Figure 3:**
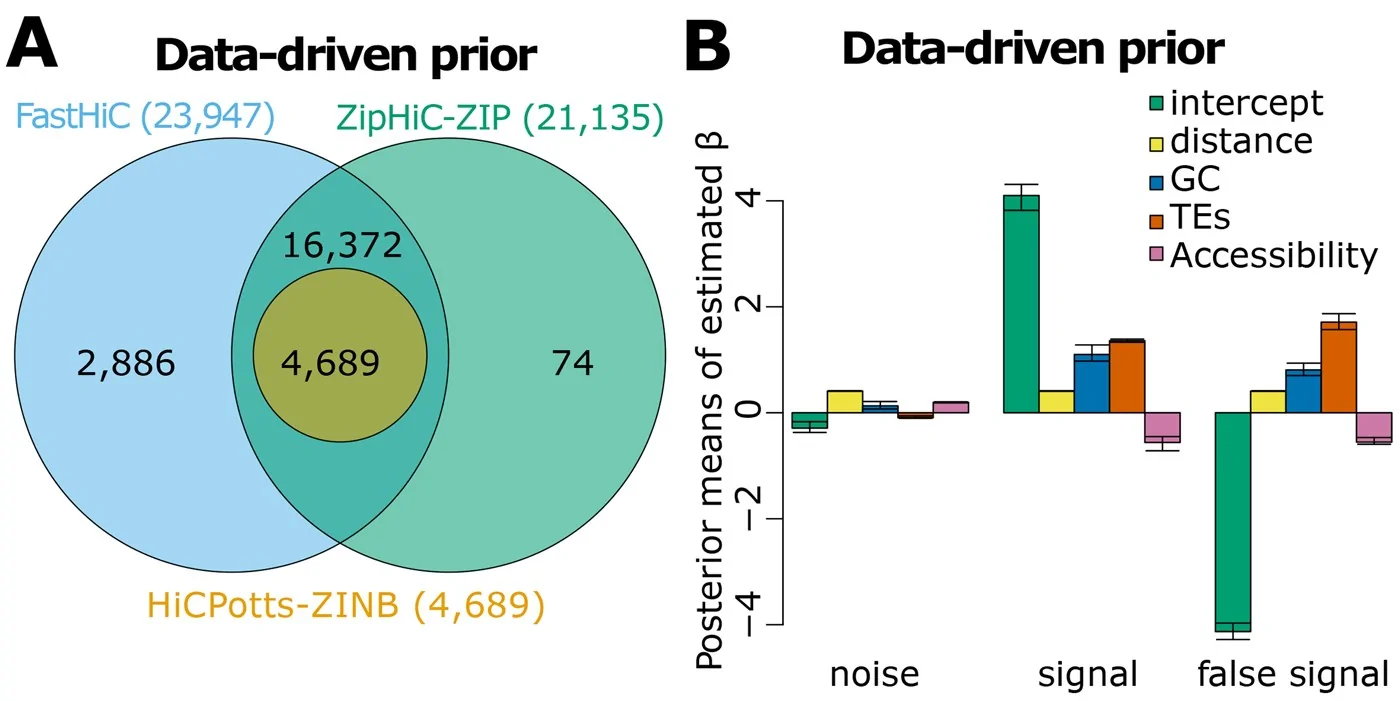
Comparison between HiCPotts (using ZINB and data-driven priors), ZipHiC and FastHiC. (A) Venn Diagram showing the comparison between HiCPotts (ZINB and data-driven priors), ZipHiC (ZIP) and FastHiC. The significant interactions were detected on a subregion of chromosome 2 L in *Drosophila* Kc167 cells. We considered that two significant interactions detected by the different tools are common if both anchors overlap fully, that is, the start and end of an anchor in one pair matches the start and end of the corresponding anchor in the other pair. The parameters for detecting the significant interactions can be found in the *Materials and Methods* section. (B) Posterior means of our estimated βs in the case of data-driven priors. We computed the posterior means as shown in Equation (3) for noise, signal and false signal components. The 95% credible intervals are shown.

First, we evaluated the significant interactions detected by HiCPotts and compared them to ZipHiC a similar method that used zero-inflated Poisson distribution (thus, not modelling the over-dispersion) and FastHiC another, hidden Markov random field based method that does not model explicitly the sources of bias (such as DNA accessibility, GC content or Transposable Elements). Figure 3A compares the significant interactions detected by HiCPotts (ZINB), ZipHiC, and FastHiC within a region of *Drosophila* chromosome 2 L. When HiCPotts uses an empirical data-driven prior, FastHiC identifies the largest number of interactions, followed by ZipHiC, while HiCPotts detects the smallest set (see Fig. 3A). Despite this difference in total counts, all HiCPotts significant interactions are a subset of the overlap between ZipHiC and FastHiC. Our results show that adding sources of bias leads to losing approximately 3K interactions and further replacing the zero-inflated Poisson distribution with a zero-inflated Negative Binomial leads to losing an additional 17K significant interactions, with only approximately 5K interactions remaining detected by all three methods. Overall, we find that the ZINB is most stringent in detecting significant interactions and has a lower rate of false positives.

A similar pattern is observed in Fig. S2A, available as supplementary data at *Bioinformatics* online, where HiCPotts is run with a user-defined prior. The number of HiCPotts-specific interactions changes only modestly between the two prior settings; see Fig. S2B, available as supplementary data at *Bioinformatics* online. 3560 (64.5%) were recovered under both, corresponding to 81.1% of the user-defined prior set and 75.9% of the data-driven set. This substantial overlap indicates that the core set of significant interactions identified by HiCPotts-ZINB is robust to prior specification and is driven primarily by the likelihood rather than the prior. This robustness is consistent with the empirical Bayes procedure described in Section *Materials and Methods*, in which the prior hyperparameters for β are re-estimated from the same data used in the likelihood at every iteration; despite this shared dependence on the data, the resulting significant interaction calls remain largely unaffected by whether the empirical or the user-defined prior is used. The remaining 1961 prior-specific interactions (832 user-defined only, 1129 data-driven only) are concentrated near the posterior-probability decision boundary ($P(z_{s}=\mathrm{signal}|y)\approx 0.90$) and therefore reflect the expected sensitivity of any threshold-based classifier to small shifts in the posterior. The consistent and extensive overlap between HiCPotts and the other two methods across both sets of priors shows that HiCPotts with ZINB recovers a stringent subset of biologically meaningful 3D interactions.

To better understand the contributions of different components to the model and identify sources of bias, we analysed the posterior means of our estimated βs of the noise, signal and false signal for the data-driven priors; see Fig. 3B. Negative values indicate that the corresponding component (noise, signal or false signal) is negatively correlated with the different features, while positive values indicate that the components are positively correlated with the different features. First, we observe that the β value for TEs on the false signal is high, indicating that regions with a higher number of TEs are more likely to be labelled as false signal by the HiCPotts model. Accessibility content is anticorrelated with the false signal and true signal. This means that some regions with lower accessibility tend to be labelled as false signal, while other regions with lower accessibility tend to be labelled as true signal by the model. Thus, the model is detecting a large number of contacts between silenced parts of the chromatin, either constitutive heterochromatin or Polycomb regions, and labels some of these as true signals while others as false signals. Polycomb regions have been shown to contribute to 3D contacts in Drosophila Ogiyama *et al.* (2018), supporting that some subset of the inaccessible parts of the genomes would display a high mean signal. Finally, GC content is lowly positively correlated with true signal and false signal, indicating potential bias from sequencing that affects the Hi-C data with regions with high GC content.

### 3.3 Identification of significant interactions in medium resolution Hi-C data micro-C data in human ES cells

**Figure 4:**
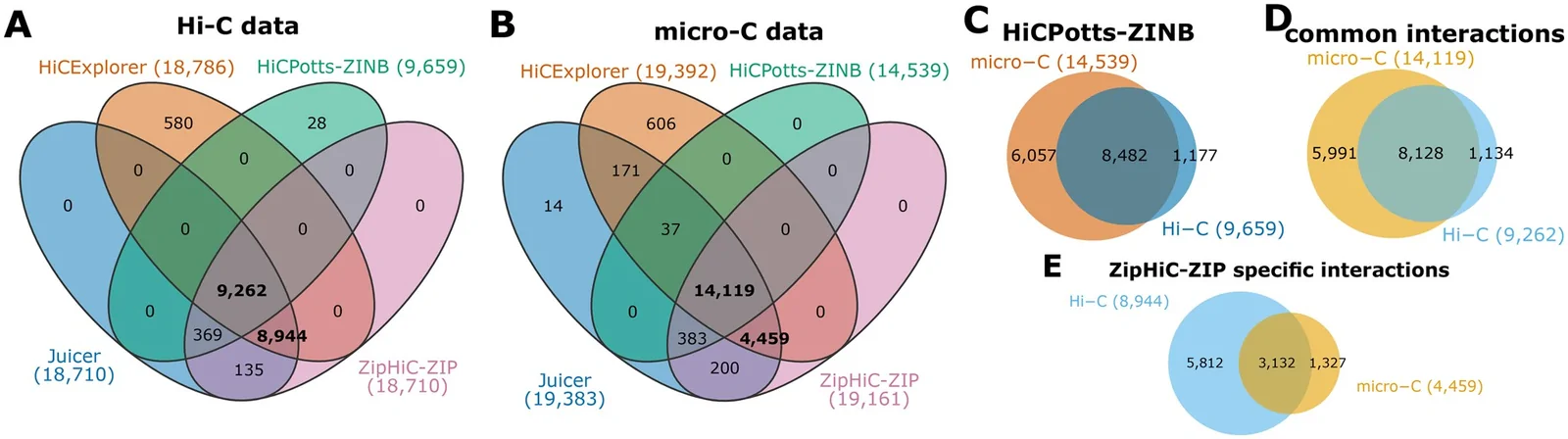
Significant interactions identified by HiCPotts and other methods on micro-C and Hi-C data in human ES cells. We consider a region of human chromosome 8 (60–70 Mb). Two interactions detected by the different tools are common if both anchors overlap fully. The parameters for detecting the significant interactions can be found in the *Materials and Methods* section. (A,B) Interactions detected by the four methods in the (A) Hi-C data and (B) micro-C data. (C) Overlap of the interactions detected by HiCPotts in the Hi-C and micro-C data. (D,E) Overlap of the interactions detected in the Hi-C and micro-C data by (D) both HiCPotts and ZipHiC and (E) ZipHiC only.

Next, we evaluated the performance of the method to identify significant interaction in Hi-C and micro-C data in human ES cells. In particular, we focused on the 60−70 Mb region of human chromosome 8 and used a 10 Kb resolution to compare HiCPotts (ZINB) to ZipHiC (using zero-inflated Poisson distribution) and two popular tools, HiCExplorer (Ramirez *et al.* 2018) and Juicer (Durand *et al.* 2016). Our results showed that all tools detect approximately 19*K* significant interactions for both Hi-C and micro-C; see Fig. 4A and B. As observed previously, HiCPotts detects significantly less interactions that are a subset of the common interactions identified by the three other methods.

Interestingly, we observe that HiCPotts detects approximately half of the interactions detected by other methods in the Hi-C data and approximately three-quarters in the micro-C data. To further dissect the differences between Hi-C and micro-C data, we compared the overlap of the significant interactions detected by HiCPotts in the Hi-C and micro-C data. Our results showed that almost all significant interactions detected in the Hi-C data are also detected in the micro-C data, but HiCPotts identified 6000 additional interactions in the micro-C; see Fig. 4C. In micro-C, the chromatin is fragmented to mono-nucleosomes using micrococcal nuclease (MNase), which increases fragment density. These results indicate that although micro-C recovers many of the Hi-C interactions, it also identifies a substantially larger set of unique significant interactions, likely reflecting the increased resolution and denser fragmentation achieved through MNase-based mono-nucleosome fragmentation compared to restriction-enzyme fragmentation in Hi-C.

We labelled as ZipHiC specific interactions, interactions that are not detected in the Hi-C or micro-C data by HiCPotts but are detected by ZipHiC, Juicer and HiCExplorer 4D-E. Figure 4D shows interactions common to Hi-C and micro-C that are detected by both HiCPotts and ZipHiC, while Fig. 4E shows the corresponding ZipHiC-only interactions. In the case of ZipHiC specific interactions, we find the opposite, namely that approximately 6000 interactions are only detected in the Hi-C data, and 3000 are detected in both Hi-C and micro-C data. Given that micro-C data has increased resolution and denser fragmentation suggests that some of these interactions that HiCPotts does not identify are likely false positives.

We further investigated the false signal interactions detected by HiCPotts-ZINB and found that 1, 671 were identified as being significant by Juicer, HiCExplorer and ZipHiC-ZIP in the micro-C data and 414 in the Hi-C data, indicating that a small subset of the contacts recovered by the other methods is being flagged as biased background by our model; see Fig. S3, available as supplementary data at *Bioinformatics* online. Consistent with the enrichment of ZipHiC-ZIP-specific calls for short-range contacts and their correlation with DNA accessibility and TE content, these shared false-signal interactions likely reflect uncorrected bias rather than genuine 3D contacts, supporting the advantage of overdispersion-aware modelling for distinguishing true signal from systematic noise.

**Figure 5:**
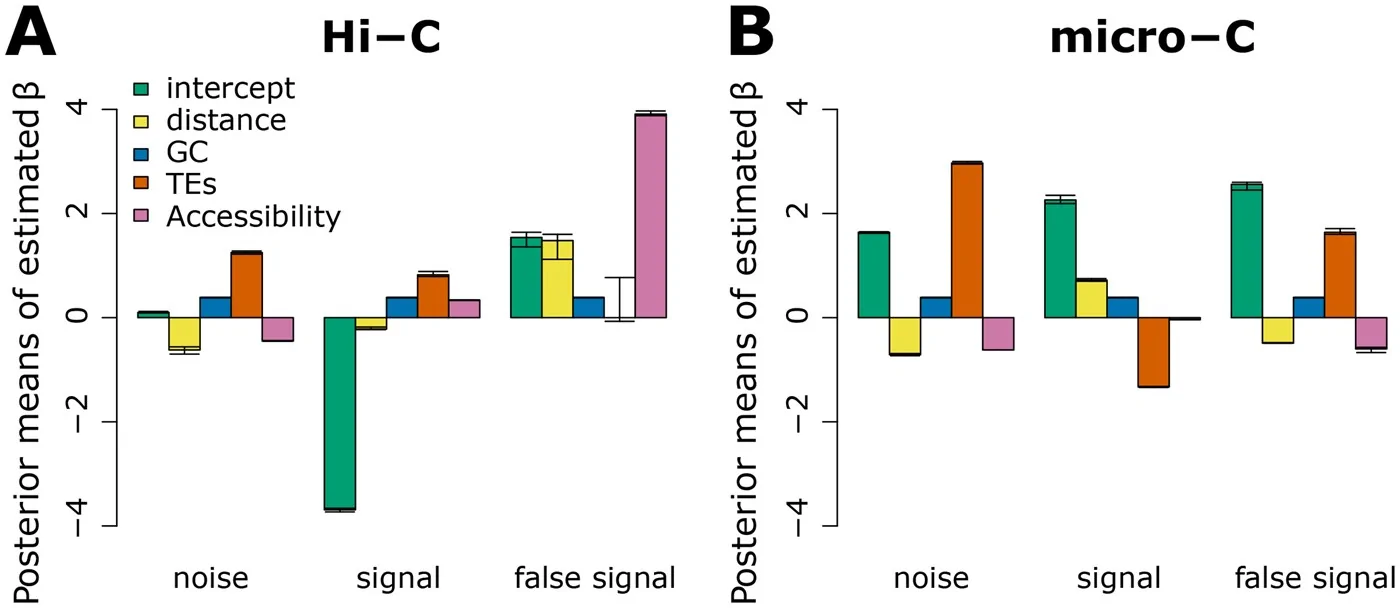
Posterior means of our estimated βs. We computed the posterior means as shown in Equation (3) for noise, signal and false signal components of human Chromosome 8, region 60, 000, 000: 70, 000, 000 for (A) Hi-C and (B) micro-C data. The 95% credible intervals are shown.

Similarly, as in the case of the *D. melanogaster* Hi-C data, we analysed the posterior means of our estimated βs of the noise, signal and false signal for both micro-C and Hi-C data; see Fig. 5. Interestingly, we observe that the DNA accessibility has a high β for the false signal component in the Hi-C data. This indicates that regions with higher accessibility are more likely to be labelled as false signal by HiCPotts model in the Hi-C data. The opposite is observed in the micro-C data, where more accessible regions are less likely to be labelled as false signal. In micro-C data, TE content seems to be correlated with false signal and noise and negatively correlated with the true signal. This means that regions with the highest TE content tend to be labelled more as false signal or noise and less as true signal by the model. Finally, GC content is lowly positively correlated with noise, true signal and false signal in both Hi-C and micro-C data. This suggests a potential modest bias from sequencing that affects both Hi-C and micro-C data similarly.

**Figure 6:**
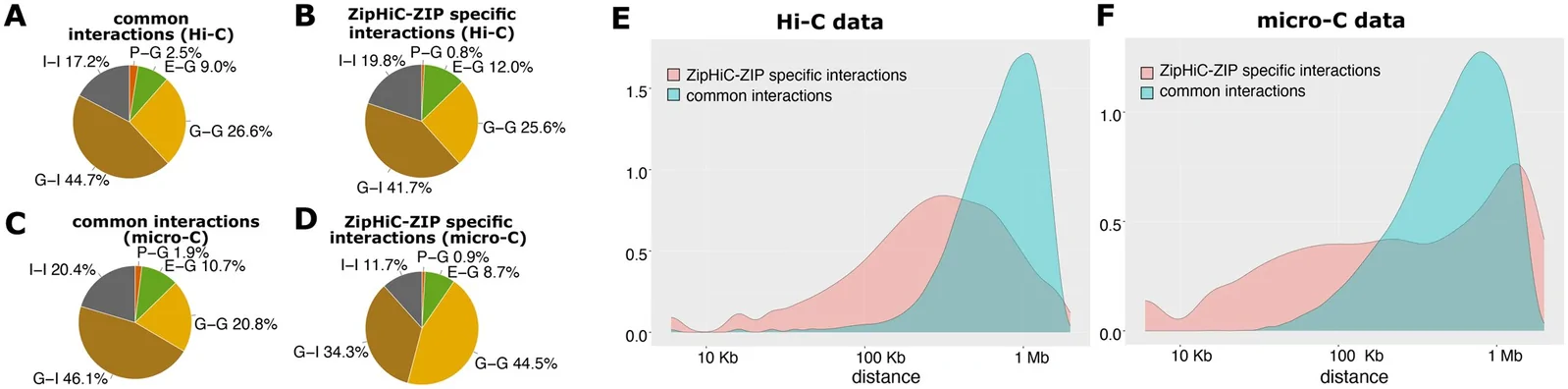
Characterisation of common and ZipHiC specific interactions in Hi-C and micro-C. (A–D) Distribution of genome annotation for the interactions identified in the 60–70 *Mb* region of human chromosome 8 by Hi-C and micro-C. Annotation of the regions linked by an interaction considers: (P) promoters (up to 1 *kb* upstream of TSS), (E) enhancers, (G) genes, TE transposable elements, and (I) Intergenic. (E,F) Distance between the two interacting regions for the (E) Hi-C and (F) micro-C data. We considered separately common (detected by HiCPotts-ZINB) and ZipHiC specific interactions.

We further characterised the significant interactions detected by all methods (common interactions) and the ones not identified by HiCPotts but identified by all other methods (ZipHiC-ZIP specific). Figure 6A–D categorises the interactions according to functional annotations. For Hi-C data, HiCPotts-ZINB detects a higher proportion of interactions linking genes and intergenic regions (including TEs or regulatory regions) or promoters, while ZipHiC-ZIP specific interactions display a higher proportion of interactions between genes and enhancers (see Fig. 6A and B). Similar results are observed for the micro-C data (see Fig. 6C and D). Nevertheless, these differences are relatively small (e.g. for enhancer gene interactions, there are 9% in the case of HiCPotts-ZINB interactions and 12% of interactions in the case of ZipHiC-ZIP specific interactions) and do not indicate that these two approaches detect functionally different groups of interactions.

Interestingly, when investigating the distance between the two interacting regions, we found that HiCPotts-ZINB detects preferentially only long-range interactions (≥100 Kb), while ZipHiC-ZIP detects both long (≥100 Kb) and short (<100 Kb) range interactions. Given that the resolution of the data is 10 Kb, interactions within 100 Kb distance are separated by less than 10 bins. Thus, these short-range interactions are more likely to be false positives. To understand whether combining HiCPotts and ZipHiC calls could improve recovery of known regulatory interactions, we constructed the combined interactions from the two methods and compared them, together with the method-specific and common subsets, against the same promoter and enhancer annotations used for the functional-annotation analysis of Fig. 6A and D (see *Supplementary Material*, Fig. S4, available as supplementary data at *Bioinformatics* online). Due to the fact that 99.7% of HiCPotts interactions are also called by ZipHiC, the combined interactions are numerically close to ZipHiC alone (18, 738 vs. 18, 710 interactions for Hi-C; 19, 198 vs. 19, 161 for micro-C). Consistent with the pattern above, the interactions uniquely detected by ZipHiC were significantly enriched for short-range contacts relative to HiCPotts overall (Hi-C: 17.3% vs. 1.6% below 100 Kb; micro-C: 28.6% vs. 2.8%).

Combining the two sets of interactions, however, did not consistently improve recovery of known regulatory interactions. For Hi-C, the ZipHiC-only interactions had a modestly higher promoter/enhancer overlap rate than HiCPotts (38.6% vs. 36.3%), and the combined interactions showed a small increase over HiCPotts alone (37.4% vs. 36.3%). For micro-C, the pattern reversed: the ZipHiC-only interactions had a substantially lower overlap with promoters and enhancers compared to HiCPotts (29.5% vs. 39.5%), and the combined interactions showed a small but significant decrease relative to HiCPotts alone (37.1% vs. 39.5%). This asymmetry between the two assays is consistent with the resolution and fragmentation differences described above: the increased resolution and denser MNase-based fragmentation of micro-C already allow HiCPotts to recover a substantially larger and more comprehensive set of significant interactions in this assay, including fine-scale promoter-enhancer contacts, so the short-range interactions additionally detected by ZipHiC in micro-C are disproportionately depleted for regulatory annotation and likely represent noise rather than missed true signal. Overall, these results indicate that the additional, predominantly short-range interactions contributed by ZipHiC do not reliably represent an enrichment for genuine regulatory contacts.

Furthermore, to quantify the overdispersion motivating the ZINB distribution model, we examined the posterior estimates of the noise-component dispersion parameter ω. Across all our datasets, ω was well below 1, confirming substantial overdispersion relative to a Poisson noise model in all three datasets and both species (see *Supplementary Material*, Table S6, available as supplementary data at *Bioinformatics* online). Comparing the magnitude of overdispersion to the size of the ZIP-to-ZINB reduction in detected interactions (see *Supplementary Material*, Table S2, available as supplementary data at *Bioinformatics* online), the dataset with the strongest overdispersion (*Drosophila*, user-defined prior) also showed the largest reduction (approximately 77%), while the dataset with the mildest overdispersion (human micro-C) showed the smallest (approximately 24%); human Hi-C was intermediate on both measures. The observed result is consistent with greater noise overdispersion driving a larger correction when replacing the Poisson-based ZIP model with ZINB.

Finally, to investigate the effect of the resolution of the data on detecting significant interactions with HiCPotts-ZINB, we analysed the Hi-C data at same 10 Mb region of human chromosome 8 using a 20 Kb resolution. Interestingly, our results showed that ZINB component posteriors for the signal call rate roughly halved (see Fig. S5, available as supplementary data at *Bioinformatics* online). This demonstrates that the call is resolution dependent.

## 4 Discussion

In this paper, we introduced a new R/Bioconductor package, HiCPotts, that can detect significant interactions in Hi-C, micro-C or capture-C data. HiCPotts is a more comprehensive version of the ZipHiC method Osuntoki *et al.* (2022) and can model contact frequencies using a Zero-Inflated Negative Binomial (ZINB) distribution, a Zero-Inflated Poisson (ZipHiC) distribution, a Poisson distribution, and a Negative Binomial (NB) distribution. HiCPotts incorporates a Potts-model hidden Markov random field (HMRF) prior to account for spatial dependencies in the Hi-C contact matrix, similar to Osuntoki *et al.* (2022), but with improved variance modelling for overdispersion. Similar to ZipHiC Osuntoki *et al.* (2022), we adopt a likelihood-free ABC approach to overcome the intractable normalising constant of the Potts model and allow a three-component mixture (*K* = 3). This allows identification of false signals, along with noise and the true signal, in the data when present. In the current implementation, (*K*) is fixed at three components to preserve a clear biological interpretation and to align with the noise, signal, and false-signal decomposition adopted in ZipHiC.

The simulation study shows that the fixed three-component assignment can adapt effectively to datasets with an underlying two-component structure. When data were generated from noise and signal components only, with no false-signal component in the true generative model, the posterior mean probability of the absent third component remained below 0.5 across all cells in the contact matrix, with the true values of the two components well estimated. Consequently, no cell in the contact matrix was assigned to that component under posterior classification, and the fitted model operated effectively as a two-component model. This finding supports application to real datasets in which only two biological components may be present, because an unsupported component is unlikely to receive a confident posterior assignment.

To investigate the most accurate distribution to model Hi-C or micro-C data, we model the contact frequencies using a Zero-Inflated Negative Binomial (ZINB) distribution, which captures the overdispersion characteristic of chromatin-contact data. Analyses of real datasets demonstrate that ZINB distribution achieves improved separation of noise and signal compared to existing tools. In particular, using the ZINB, we detect only a subset of the interactions detected by ZIP, namely approximately half for Hi-C and three-quarters for micro-C.

The noticeable difference in the number of significant interactions detected by the HiCPotts (ZINB) and the ZipHiC reflects the distinct statistical assumptions underlying the methods. HiCPotts (ZINB), which models the background using a zero-inflated Negative Binomial distribution and explicitly accounts for overdispersion, detects fewer significant interactions than ZipHiC, which uses a zero-inflated Poisson model. The HiCPotts (ZINB) model allows for a larger variance in the noise component, which leads to moderately high contact counts being accounted as part of a highly variable background rather than being attributed to the signal component. In contrast, the Poisson-based ZipHiC model underestimates the variance, making the same counts appear more extreme, and thus more likely to be classified as signal. This leads to a more conservative set of calls for HiCPotts (ZINB), enriched for robust interactions, and a larger set of calls for ZipHiC that includes the same interactions as HiCPotts (ZINB), but also additional, more borderline counts.

The interactions detected by HiCPotts-ZINB display a higher proportion of genes to regulatory regions interactions (intergenic, promoters or enhancers) than the interactions detected only by ZipHiC-ZIP. Most importantly, HiCPotts-ZINB preferentially detects only significant interactions beyond 100 Kb, while the interactions detected only by ZipHiC-ZIP are mostly between 10 Kb and 100 Kb. Previous work has shown that *cis*-regulatory regions control gene activity within 50 Kb, while, for longer distances, 3D contacts might be required Guckelberger *et al.* (2024), although that might not always be the case Benabdallah *et al.* (2019). This suggest that these shorter range interactions detected only by ZipHiC-ZIP might not only be false positives, but also might not have a functional role if they do exist. Furthermore, when combining HiCPotts and ZipHiC interactions, the combined interactions did not show a consistent gain in promoter/enhancer overlap over HiCPotts alone in the case of Hi-C data, and were substantially lower for the micro-C data. This indicates that the additional short-range interactions uniquely detected by ZipHiC do not represent a gain in biologically meaningful signal, further supporting the interpretation that HiCPotts-ZINB more conservative calls are enriched for genuine 3D contacts rather than omitting complementary true positives.

This distance-dependent pattern is likely influenced by the resolution used in this analysis (10 Kb), the number of bins spanning a given genomic distance, and hence the number of neighbouring bins available to the Potts spatial prior, scales with resolution. Interestingly, we showed that using a lower resolution (20 Kb) leads to a reduction by half of the detected significant interactions. At a higher resolution, such as 5 Kb, the same 100 Kb region would be represented by twice as many bins, which could allow the spatial smoothing in HiCPotts to borrow more information across neighbouring short-range interactions and potentially recover more of them as genuine signal rather than noise. Conversely, higher-resolution data typically have sparser per-bin contact counts and greater zero-inflation, which could instead reinforce the current pattern of preferentially detecting long-range interactions.

While investigating the mixture components of the HiCPotts-ZINB model in human ES cells, we identified a high correlation between DNA accessibility and false signal in the Hi-C data with regions with higher accessibility being more likely to be labelled as false signal by our model. In addition, we also found that regions with higher TE content tend to be labelled more as false signal or noise and less as true signal in micro-C data. These results highlight the importance of including known potential sources of bias when modelling Hi-C or micro-C data.

While HiCPotts models additional features, including the overdispersion effect, spatial structure and multiple mixture components, that are not captured by other methods, this comes at a computational cost. HiCPotts, specifically when using the ZINB, is particularly well suited for Capture-C or targeted regions of Hi-C or micro-C datasets, where the number of bins is smaller, and the spatial structure can be exploited fully without heavy runtime. While Hi-C and micro-C capture chromatin interactions genome-wide, Capture-C is inherently region-specific, enriching only for interactions within a targeted subset of the genome anchored at chosen viewpoints, and can therefore be regarded as a spatially restricted subset of Hi-C data. Because HiCPotts can operates on an arbitrary, user-defined genomic interval as well as genome-wide input, it is directly applicable to Capture-C targeted datasets.

In conclusion, HiCPotts offers a flexible and powerful framework for identifying significant chromatin interactions in Hi-C or micro-C data. By accounting for multiple assumptions for the contact frequencies distribution, options for user-specified priors, jointly modelling overdispersion, spatial structure, and multiple latent components, it distinguishes noise from true signal more accurately than existing methods and provides insight into the sources and magnitudes of experimental bias. Our results demonstrate that HiCPotts is a robust tool for dissecting and understanding the complex structure of 3D genome architecture.

## Supplementary Material

btag673_Supplementary_Data
