# Supplementary material for: HiCPotts: an R/bioconductor package to identify significant interactions in chromosome conformation capture data and model sources of bias
Source: Bioinformatics. 2026 Sep 10;42(9):btag673. doi: 10.1093/bioinformatics/btag673 (PMC13612170; doi:10.1093/bioinformatics/btag673)
Supplement: Supplementary file 1 [file btag673_supplementary_data.pdf]

# Supplementary Material to *HiCPotts: An R/Bioconductor package to identify significant interactions in chromosome conformation capture data and model sources of bias.*

Itunu Godwin Osuntoki[1,2,*] Andrew Harrison[2] Hongsheng Dai[3] Yanchun Baoi[2] and Nicolae Radu Zabet[4,5,*]

[1]Statistics, Modelling and Economics Department, UK Health Security Agency, 61 Colindale Ave, London, NW9 5EQ, United Kingdom; [2]School of Mathematics, Statistics and Actuarial Science, University of Essex,Wivenhoe Park, Colchester, CO4 3SQ, United Kingdom; [3]School of Mathematics, Statistics and Physics, Newcastle University, Newcastle upon Tyne, NE1 7RU, United Kingdom; [4]Blizzard Institute, Barts and The London School of Medicine and Dentistry, Queen Mary University of London, London, E1 2AT, United Kingdom; [5]Centre for Epigenetics, Queen Mary University of London, London, E1 2AT, United Kingdom

[*] Corresponding authors: hitunes4@gmail.com, r.zabet@qmul.ac.uk

## S1 Model for Data

The complete likelihood function of the unknown parameters $(\boldsymbol{\beta}, \boldsymbol{\alpha}, \boldsymbol{\tau})$ given the data $y$ and labels $z$ can then be written as

$$l(\vec{\boldsymbol{\beta}}, \vec{\boldsymbol{\omega}}, \boldsymbol{\alpha}, \boldsymbol{\tau} \mid \boldsymbol{y}, \boldsymbol{z}) \propto \prod_{i=1}^{n}\prod_{j=1}^{n}\Bigg\{ \Big[\alpha_1\Big(\tau\, \mathbb{I}\{y_{ij}=0\} + (1-\tau)\,\mathrm{NB}\Big(y_{ij}\,;\, \lambda_{ij}^{(1)},\, \omega^{(1)}\Big)\Big)\Big]^{\mathbb{I}(z_{ij}=1)} \prod_{k=2}^{K}\Big[\alpha_k\,\mathrm{NB}\Big(y_{ij}\,;\,\lambda_{ij}^{(k)},\,\omega^{(k)}\Big)\Big]^{\mathbb{I}(z_{ij}=k)}\Bigg\}, \tag{S1}$$

where $\mathrm{NB}\Big(y;\lambda,\omega\Big) = \frac{\Gamma(y+\omega)}{\Gamma(\omega)\,y!}\left(\frac{\omega}{\omega+\lambda}\right)^{\omega}\left(\frac{\lambda}{\omega+\lambda}\right)^{y}, \quad y \in \{0,1,2,\ldots\},$

The full posterior of $z$, $\vec{\beta}$ and $\gamma$ given $y_{ij}$ is

$$Pr(\boldsymbol{z}, \vec{\boldsymbol{\beta}}, \gamma|\boldsymbol{y}) \propto l(\vec{\boldsymbol{\beta}}, \boldsymbol{\alpha}, \boldsymbol{\tau}|\boldsymbol{y}, \boldsymbol{z})\, l(\boldsymbol{z}|\gamma)\pi_0(\gamma)\tau_0(\vec{\boldsymbol{\beta}}) \tag{S2}$$

where $l(\boldsymbol{z}|\gamma) = \dfrac{e^{\gamma\sum_{s\sim t}\delta_{z_s z_t}}}{\sum_{z_s} e^{\gamma\sum_{s\sim t}\delta_{z_s z_t}}}$ is the Potts model, $s$ is bin pair $i$ and $j$, and $t$ is the neighbours set of $s$; $(i-1,\, i+1,\, j-1,\, j+1)$.

In order to analyse our data and estimate our parameters, we make use of the Metropolis-within-Gibbs sampler and the Approximate Bayesian Computation (ABC), so the conditional posterior densities are needed.

### S1.1 Conditional Posterior Density

The conditional posterior of $\tau$ is given as

$$Pr(\tau|\vec{\boldsymbol{\beta}}^{\mathbf{1}}, \boldsymbol{y}, \boldsymbol{z}) \propto \prod_{i=1}^{n}\prod_{j=1}^{n}[f^{(1)}(y_{ij}; \boldsymbol{\beta}^{(1)})]^{I[z_{ij}=1]} \cdot \pi_0(\tau) \tag{S3}$$

The conditional posterior of $\beta$ is given by

$$Pr(\vec{\boldsymbol{\beta}}^{(\boldsymbol{k})}|\boldsymbol{y}, \boldsymbol{z}) \propto \prod_{i=1}^{n}\prod_{j=1}^{n}\prod_{k}^{K}[f^{(k)}(y_{ij}; \boldsymbol{\beta}^{(k)})]^{I[z_{ij}=k]}\pi_0(\boldsymbol{\beta}^{(k)}) \tag{S4}$$

Based on the definitions of $f^{(k)}(y_{ij})$ and equation S4, the conditional posterior of $\beta^{(1)}$ for the noise (ZI) component can be rewritten as

$$\Pr(\vec{\boldsymbol{\beta}}^{(\mathbf{1})} \mid \boldsymbol{y}, \boldsymbol{z}) \propto \prod_{i=1}^{n}\prod_{j=1}^{n}\Big[\Big(\tau\,\mathbb{I}\{y_{ij}=0\} + (1-\tau)\,\mathrm{NB}\Big(y_{ij};\, \lambda_{ij}^{(1)},\, \omega^{(1)}\Big)\Big)\Big]^{\mathbb{I}(z_{ij}=1)} \exp\bigg\{-\frac{\big(\beta^{(1)}-m^{(1)}\big)^2}{2\,(\sigma^2)^{(1)}}\bigg\}. \tag{S5}$$

For the signal component, the conditional posterior of $\beta^{(k)}$ (for $k \geq 2$) based on the definition of $f^{(k)}(y_{ij})$ and equation S4 can be rewritten as

$$\Pr(\vec{\boldsymbol{\beta}}^{(\boldsymbol{k})} \mid \boldsymbol{y}, \boldsymbol{z}) \propto \prod_{i=1}^{n}\prod_{j=1}^{n}\Big[\mathrm{NB}\Big(y_{ij};\, \lambda_{ij}^{(k)},\, \omega^{(k)}\Big)\Big]^{\mathbb{I}(z_{ij}=k)} \exp\bigg\{-\frac{\big(\beta^{(k)}-m^{(k)}\big)^2}{2\,(\sigma^2)^{(k)}}\bigg\}. \tag{S6}$$

where $m^{(k)}$ is the mean and $\big(\sigma^{(k)}\big)^2$ is the variance for component $k$. Here $\mathrm{NB}(y;\lambda,r)$ denotes the negative binomial pmf parameterised by mean $\lambda$ and size $r$, and $\lambda_{ij}^{(k)} = \exp\big(x_{ij}^{\top}\beta^{(k)}\big)$ under the log link.

The conditional posterior of the NB size parameter ($\omega$) is given as

$$\Pr\Big(\omega^{(1)} \mid \vec{\boldsymbol{\beta}}^{(1)}, \tau, \boldsymbol{y}, \boldsymbol{z}\Big) \;\propto\; \prod_{i=1}^{n}\prod_{j=1}^{n}\Big[\, f^{(1)}\Big(y_{ij};\, \boldsymbol{\beta}^{(1)}, \omega^{(1)}, \tau\Big)\Big]^{\mathbb{I}[z_{ij}=1]} \cdot \pi_0(\omega^{(1)}). \tag{S7}$$

$$\Pr\Big(\vec{\omega}^{(k)} \mid \vec{\boldsymbol{\beta}}^{(k)}, \boldsymbol{y}, \boldsymbol{z}\Big) \;\propto\; \prod_{i=1}^{n}\prod_{j=1}^{n}\Big[\, f^{(k)}\Big(y_{ij};\, \boldsymbol{\beta}^{(k)}, \omega^{(k)}\Big)\Big]^{\mathbb{I}[z_{ij}=k]} \cdot \pi_0(\omega^{(k)}). \tag{S8}$$

To update the latent variable, the probability of an observation belonging to each component is calculated

$$Pr(z_s|\gamma, z_t, \boldsymbol{y}, \vec{\boldsymbol{\beta}}^{(\boldsymbol{k})}) \propto e^{\gamma \sum_{s\sim t} \delta_{z_s z_t}} f(y_{i,j}; \boldsymbol{\beta}^{(k)}) \tag{S9}$$

where $s$ is bin pair $i$ and $j$, and $t$ is the neighbours set of $s$; ($i-1$, $i+1$, $j-1$, $j+1$) and $f^{(k)}(y_{ij}; \vec{\boldsymbol{\beta}}^{(\boldsymbol{k})})$ is the likelihood of component $k$.

When the normalizing constant is introduced, equation S9 can be rewritten as

$$Pr(z|\gamma, z_t, \boldsymbol{y}, \vec{\boldsymbol{\beta}}^{(k)}) = \frac{e^{\gamma \sum_{s\sim t} \delta_{z_s z_t}}}{\sum_{z_s} e^{\gamma \sum_{s\sim t} \delta_{z_s z_t}}} \frac{f(y_{i,j}; \boldsymbol{\beta}^{(k)})}{f(y_{i,j}; \boldsymbol{\beta}^{(k)})} \tag{S10}$$

where $s$ is bin pair $i$ and $j$, and $t$ is the neighbour(s).

The conditional probability of $\gamma$ in the Potts model is given as

$$Pr(\gamma|\boldsymbol{y}, \boldsymbol{z}, \vec{\boldsymbol{\beta}}) = \frac{\exp\{\gamma \sum_{s\sim t} \delta(z_s z_t)\}\pi_0(\gamma)}{\sum_{z_s} \exp\{\gamma \sum_{s\sim t} \delta(z_s z_t)\}\pi_0(\gamma)} \tag{S11}$$

Algorithm 1: ABC

```
repeat
    Select the initial value of γ^0;
    m = 0
    for i = 1 : N do
        Compute a new y* based on the Potts model and updated β⃗ from Algorithm (2)
        Compute the distance d(S(y*), S(y))
        Select ϵ using 1% empirical quantile of the distance or other chosen distance metric
        if d(S(y*), S(y)) < ϵ then
            γ_{m+1} = (γ_m)*
        end ifend if
    end forend for
until enough MCMC steps have been simulated
```

[1]

Algorithm 2: Metropolis-within-Gibbs sampler

```
procedure
    Initialization, select initial value,z^0, γ^0, τ^0, ω^0, β⃗^0;
    repeat
        for i = 1 to n, j = 1 to n do
            Update z_ij using (S11)
        end forend for
        Update β⃗ from posterior in (S5)and (S6)
        Update τ using (S3)
        Update ω from posterior in (S7) and (S8)
        Update γ using Algorithm (1)
    until enough MCMC steps have been simulated;
end procedure
```

The equations for $\vec{\beta}$'s, $\vec{\omega}$'s and $z$ as given above in equations S3, S5, S6, S7 and S8 is computationally easier to simulate using the Metropolis-Hastings-within-Gibbs sampler. Equation S11 is computationally intractable as the interaction parameter $\gamma$ involves the evaluation of the partition function and cannot be simulated directly using the Gibbs sampler or the Metropolis-Hastings sampler.

Algorithm 1 shows the Approximate Bayesian Computation (ABC) approximate steps and Algorithm 2 shows the Metropolis-within-Gibbs steps used in this paper to update our parameters.

# S2 Simulated dataset

Table S1 shows the component dispersions under a lower-overdispersion scenario (larger size values) and a higher-overdispersion scenario (smaller size values). The components are recovered well in both, except in the low overdispersion scenario when setting ($\omega^{(3)}$) to be $40$ makes it closer to a standard Poisson distribution.

Table S1: *Dispersion parameters at two further overdispersion scenarios* (ZINB, 15 replicates).

| Scenarios | Parameter (true) | Mean estimate | 95% CI |
|---|---|---|---|
| low overdispersion | $\omega^{(1)} = 6$ | 4.87 | [1.15, 14.29] |
| | $\omega^{(2)} = 16$ | 16.48 | [9.03, 30.07] |
| | $\omega^{(3)} = 40$ | 39.14 | [22.77, 65.06] |
| high overdispersion | $\omega^{(1)} = 1.5$ | 1.62 | [0.52, 9.30] |
| | $\omega^{(2)} = 4$ | 4.79 | [2.28, 10.74] |
| | $\omega^{(3)} = 10$ | 11.10 | [7.06, 15.34] |

We also assumed different component dominance and found that HiCPotts can recover well the simulated parameters; see Figure S1A-C. To confirm that the estimation of the HiCPotts is not specific to the zero-inflated case, we repeated our simulation under the Negative Binomial distribution model (no zero-inflation). Figure S1D shows the estimated parameters.

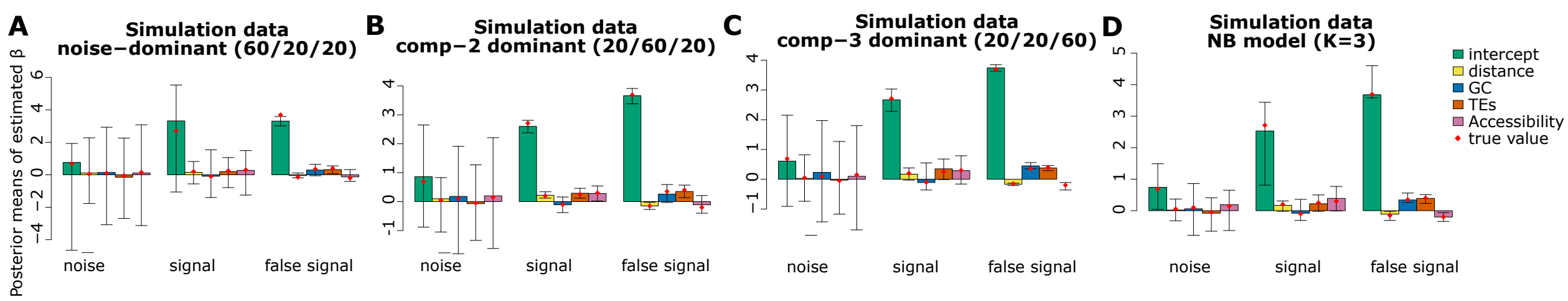


Figure S1: *Simulation studies assuming different component dominance or distributions.* Posterior means of our estimated $\beta$'s for different component dominance or distribution using simulated data: *(A)* The estimated $\beta$'s when the noise component is the dominant component; *(B)* the estimated $\beta$'s when component 2 is the dominant component; *(C)* the estimated $\beta$'s when component 3 is the dominant component; and *(D)* the estimated $\beta$'s when we assume a Negative Binomial instead of the ZINB with K = 3.We include the 95% credible interval as shown in the box plots.

## S3 Comparison between data-driven and user defined priors.

Figure S2 confirms that there are negligible differences between the results obtained using user-defined priors and data-driven priors.

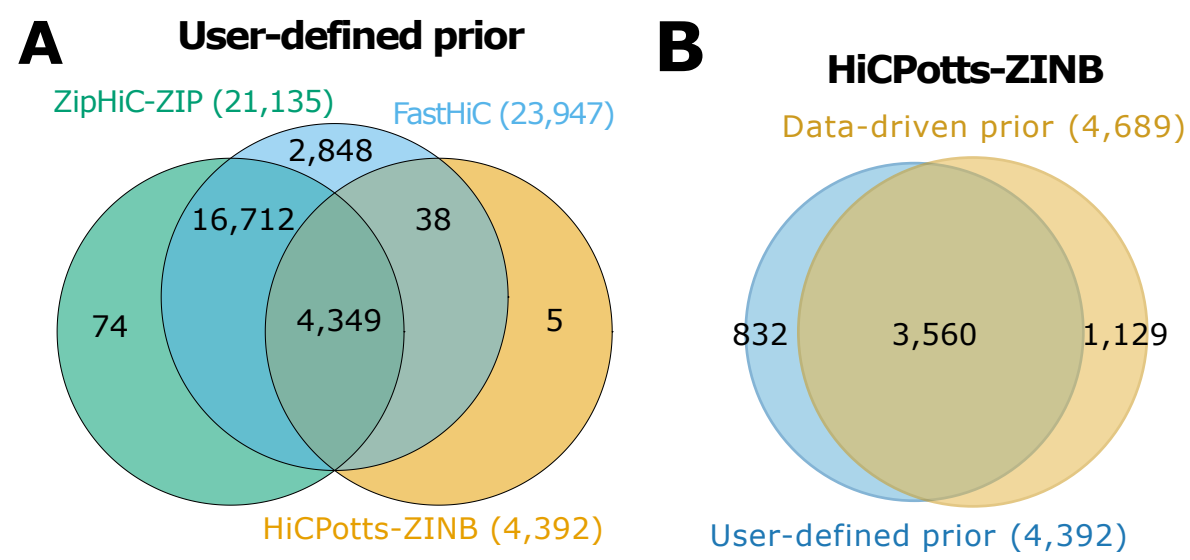


Figure S2: *Comparison between HiCPotts (using ZINB and user-defined priors), ZipHiC and FastHiC. (A)* Venn Diagram showing the comparison between HiCPotts (ZINB with user-defined priors), ZipHiC (ZIP) and FastHiC. The signficant interactions were detected on a subregion of chromosome 2L in *Drosophila* Kc167 cells. We considered that two significant interactions detected by the different tools are common if both anchors overlap fully, that is, the start and end of an anchor in one pair matches the start and end of the corresponding anchor in the other pair. The parameters for detecting the significant interactions can be found in the *Materials and Methods* section. *(B)* The overlap between the significant interactions detected by HiCPotts (using ZINB) using the two sets of priors (data-driven or user-defined).

## S4 False signal identified in human ES cells dataset

We labelled the third component (K=3) as false signal, due to its significantly high values beyond the expected values, suggesting potential technical artefacts. Figure S3 shows that almost all these interactions that were classified as false signal with ZINB, were detected as true signal when using the ZIP distribution or using other tools such as Juicer or HiCExplorer.

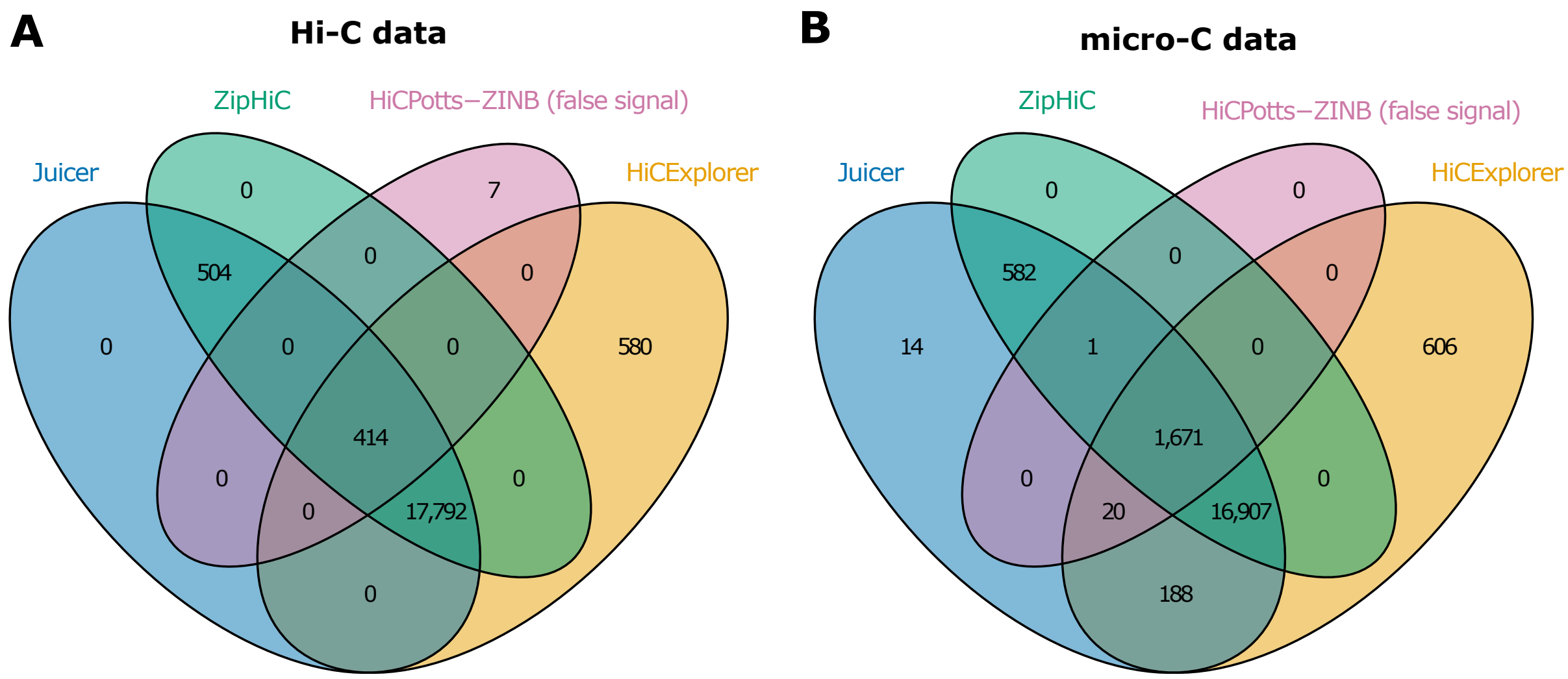


Figure S3: *Significant interactions identified by HiCPotts as false signal and as true signal by other methods on micro-C and Hi-C data in human ES cells*. We consider a region of human chromosome 8 (60-70Mb). Two interactions detected by the different tools are common if both anchors overlap fully. The parameters for detecting the significant interactions can be found in the *Materials and Methods* section. *(A-B)* Interactions detected by the four methods in the *(A)* Hi-C data and *(B)* micro-C data.

## S5 Interdependency of HiCPotts and ZipHiC calls

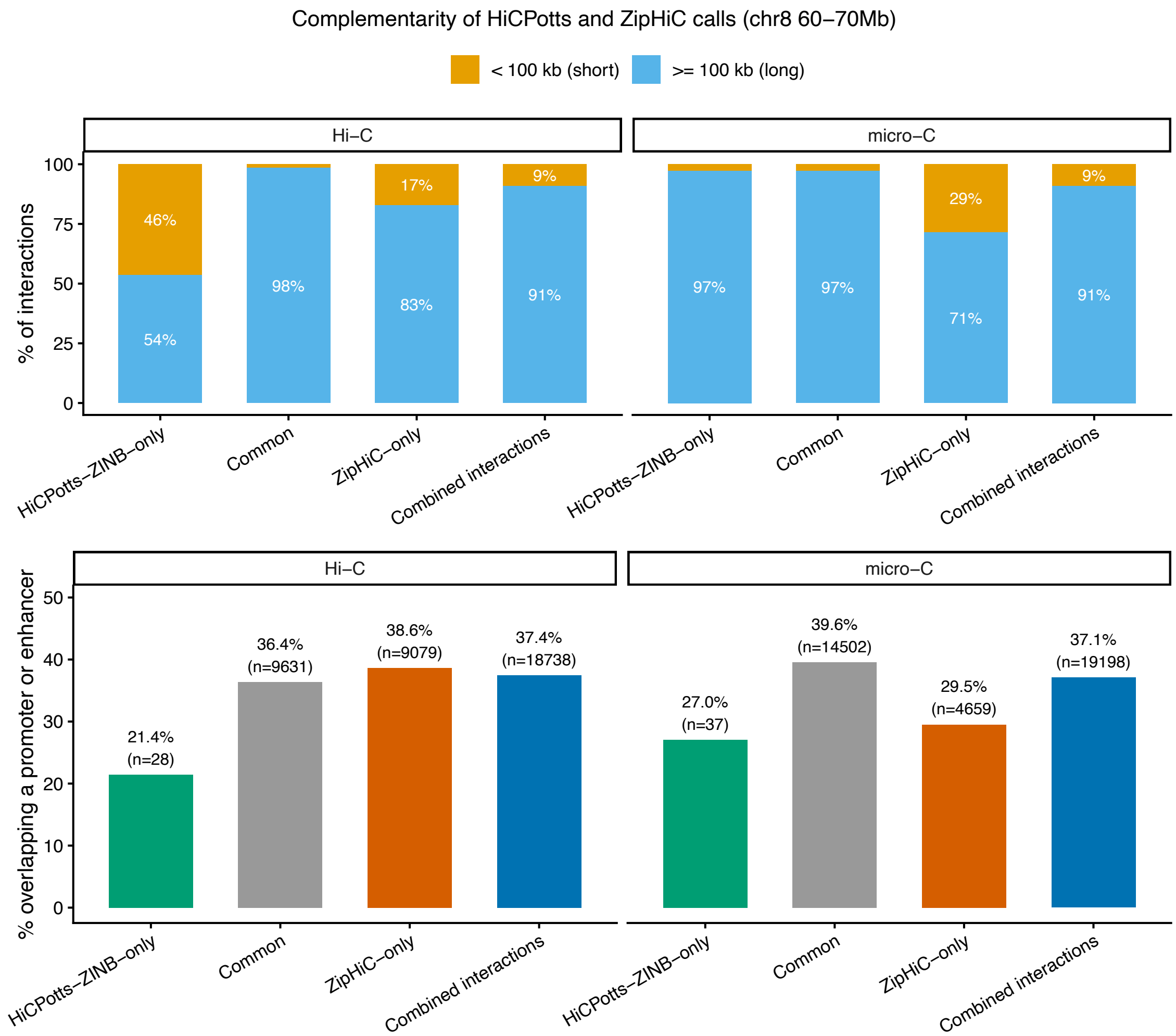


Figure S4: *Interdependency of HiCPotts-ZINB and ZipHiC calls on chr8 (60–70Mb). (Top)* Proportion of short-range ($< 100\ Kb$) and long-range ($\geq 100\ Kb$) interactions for the HiCPotts-ZINB-only, Common (detected by both), ZipHiC-only, and Combined interactions (HiCPotts $\cup$ ZipHiC) sets, for Hi-C and micro-C. *(Bottom)* Percentage of interactions in each set with at least one anchor overlapping an annotated promoter or enhancer. Sample sizes ($n$) are shown above each bar; the HiCPotts-ZINB-only category is a small sample (Hi-C: $n = 28$; micro-C: $n = 37$) and is shown for completeness.

## S6 Overdispersion real dataset

Table S2 reports the posterior mean and 95% credible interval of the noise-component's negative-binomial overdispersion parameter ($\omega$) for each dataset analysed in this study. The estimated overdispersion parameter ($\omega$) is reported independently, as it strongly confirms severe overdispersion and validates the use of the ZINB distribution. In every dataset, the ($\omega$) is well below 1, confirming substantial overdispersion relative to a Poisson noise model. The dataset with the strongest overdispersion (*Drosophila*, user-defined prior) also shows the largest ZIP-to-ZINB reduction, and the dataset with the lowest overdispersion (human micro-C) shows the smallest reduction, with human Hi-C intermediate on both measures (see *Results*).

Table S2: Noise-component overdispersion ($\omega$).

| Dataset | ($\omega$) (noise) mean | 95% CrI |
|---|---|---|
| *Drosophila* (user-defined prior) | 0.69 | (0, 5.90) |
| *Drosophila* (data-driven prior) | 0.89 | (0, 5.91) |
| Human Hi-C (chr8, 60–70 Mb) | 0.71 | (0, 5.90) |
| Human micro-C (chr8, 60–70 Mb) | 0.72 | (0, 5.80) |

## S7 Data Resolution effect on HiCPotts

At $10\ Kb$, $72.3\%$ of bin-pairs are called signal, while after aggregating to $20\ Kb$, only $37.8\%$ are called true signal (posterior $\text{prob}_2 \geq 0.5$); see Figure S5. This indicates that halving the resolution nearly halves the signal-call rate, indicating that the HiCPotts model, using the ZINB distribution, is resolution dependant.

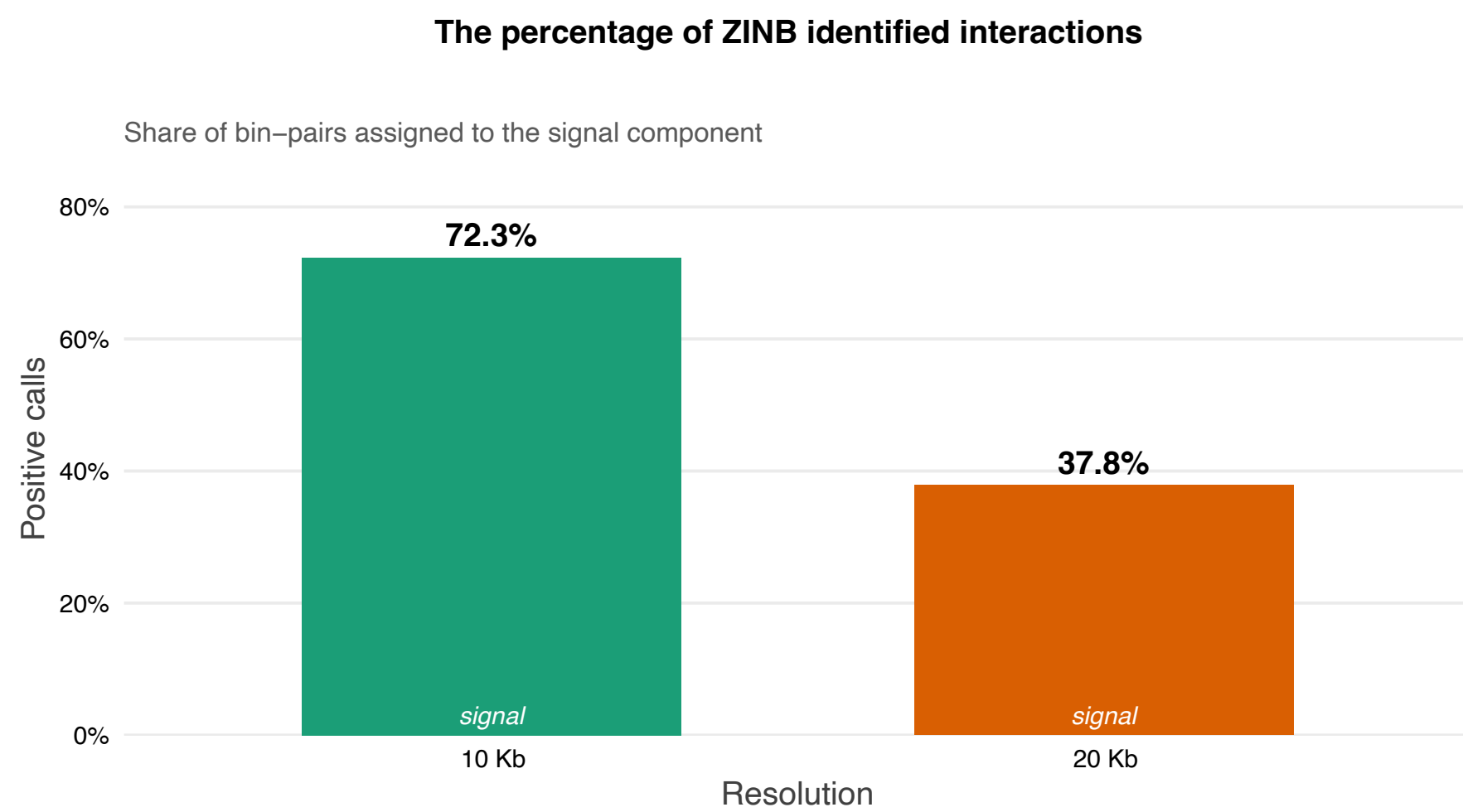


Figure S5: *HiCPotts is resolution-dependent.* Proportion of bin-pairs assigned to the ZINB signal component at $10\ Kb$ compared to a lower resolution ($20\ Kb$) on chr8 ($60 - 70\ Mb$).
